# The emerging role of B cells in immune-mediated demyelinating diseases: mechanisms and therapeutic implications

**DOI:** 10.3389/fimmu.2026.1855701

**Published:** 2026-07-08

**Authors:** Yawei Liu, Henrik Hasseldam

**Affiliations:** Biotech Research & Innovation Centre (BRIC), Faculty of Health and Medical Sciences, University of Copenhagen, Copenhagen, Denmark

**Keywords:** autoimmunity, B cells, demyelinating diseases, mutiple sclerosis, myelin oligodendrocyte (MOG) antibody associated disease, neuromyelities optica spectrum disorders

## Abstract

Immune-mediated demyelinating diseases of the central nervous system, including multiple sclerosis (MS), neuromyelitis optica spectrum disorder (NMOSD), and myelin oligodendrocyte glycoprotein antibody–associated disease (MOGAD), are unified by inflammatory injury to myelin and axons but differ fundamentally in their immunopathological mechanisms. Adaptive immune cells, particularly B cells and T cells, are central drivers of disease, contributing through both antibody-dependent and antibody-independent mechanisms. In MS, B cells exacerbate pathology via antigen presentation, cytokine secretion, and organization of ectopic lymphoid structures, consistent with the efficacy of anti-CD20 therapies despite largely preserved circulating immunoglobulin levels. In NMOSD, B cells and plasma cell progeny play a direct pathogenic role through aquaporin-4–specific (AQP-4) antibodies, establishing a model of antibody-mediated astrocytopathy. MOGAD occupies an intermediate immunopathological niche, with pathogenic antibodies targeting myelin oligodendrocyte glycoprotein (MOG) coexisting with additional, incompletely defined immune mechanisms. Recent advances from single-cell profiling, high-resolution imaging, and experimental models suggest substantial heterogeneity in B-cell developmental states, tolerance checkpoints, and tissue residency, challenging uniform approaches to B-cell–directed therapy. This review synthesizes current understanding of B-cell biology across demyelinating diseases, highlighting mechanisms of pathogenicity, immune regulation, and tolerance failure, and discusses implications for precision immunomodulatory strategies.

## Introduction

Immune-mediated demyelinating diseases of the central nervous system (CNS), including multiple sclerosis (MS), neuromyelitis optica spectrum disorder (NMOSD), and myelin oligodendrocyte glycoprotein antibody–associated disease (MOGAD), are characterized by inflammatory injury to myelin and axons that ultimately leads to neurological disability. Although these disorders share overlapping clinical and radiographic features, they are distinguished by fundamental differences in immune mechanisms and target cell vulnerability (1).

MS was historically regarded as a predominantly T cell–driven autoimmune disease (2), with B cells viewed as secondary contributors primarily through antibody production. This paradigm has shifted substantially over the past two decades. Converging evidence suggests a central role for B cells in CNS inflammation from experimental models, neuropathological studies, and clinical trials (3). The therapeutic efficacy of anti-CD20 monoclonal antibodies, including rituximab, ocrelizumab, and ofatumumab, provides compelling support for this concept. These agents robustly reduce relapse rates and disease activity despite sparing long-lived plasma cells and largely preserving circulating immunoglobulin G levels (4, 5). Within MS and related demyelinating disorders, B cells exhibit marked functional heterogeneity, acting as both drivers of pathology and mediators of immune regulation. For instance, autoreactive B cells amplify neuroinflammation through antibody production, antigen presentation, costimulatory signaling, and secretion of proinflammatory cytokines that exacerbate tissue injury (6–8). In contrast, regulatory B-cell subsets (Breg) maintain immune homeostasis through anti-inflammatory cytokines’ production and other immunomodulatory mechanisms (9, 10). Importantly, in CNS demyelinating diseases, B cells exert functions beyond antibody production, including interactions with T cells that drive T-cell activation, differentiation, and lymphoid organization. These processes are particularly relevant within the immune-privileged yet immunologically dynamic CNS (11–13).

In contrast to MS, NMOSD represents a prototypical antibody-mediated CNS autoimmune disorder. In AQP-4 IgG–positive NMOSD, B cells and their differentiated progeny play a direct pathogenic role by producing AQP4-specific antibodies that target astrocytic water channels and trigger severe astrocyte injury. This process subsequently induces secondary demyelination and neuronal death (14). Consistent with this mechanism, B-cell–targeted therapies are highly effective in NMOSD. Both anti-CD20 agents and broader anti-CD19 approaches, such as inebilizumab, markedly reduce relapse risk, underscoring the causal role of antibody-producing B-cell lineages in disease pathogenesis (15).

MOGAD occupies an intermediate position between MS and NMOSD with respect to B-cell biology (16). In MOGAD, B cells produce IgG1 antibodies directed against conformational epitopes of MOG that are expressed on the outer surface of myelin and oligodendrocytes, resulting in a primary oligodendrocytopathy (1, 15–19). Unlike NMOSD, the precise pathogenic contribution of MOG-IgG remains incompletely defined. Available experimental and clinical evidence suggests that antibody-mediated injury coexists with additional immune mechanisms, including antigen presentation, cytokine secretion, antibody-dependent cellular cytotoxicity, and broader contributions from other immune cell populations (19, 20).

Recent advances in single-cell profiling, high-resolution imaging, and experimental models have uncovered additional layers of B-cell complexity across demyelinating diseases, including distinct developmental states, defects in tolerance checkpoints, and tissue-resident B-cell populations that influence disease course (21). These insights challenge the notion that broad B-cell depletion might not be universally optimal. Instead, disease stage, anatomical niche, and B-cell functional state may also determine therapeutic response. A deeper understanding of B-cell subset heterogeneity, functional specialization, and metabolic requirements may enable more precise strategies that suppress pathogenic responses while preserving protective and regulatory functions (22).

**Figure 1:**
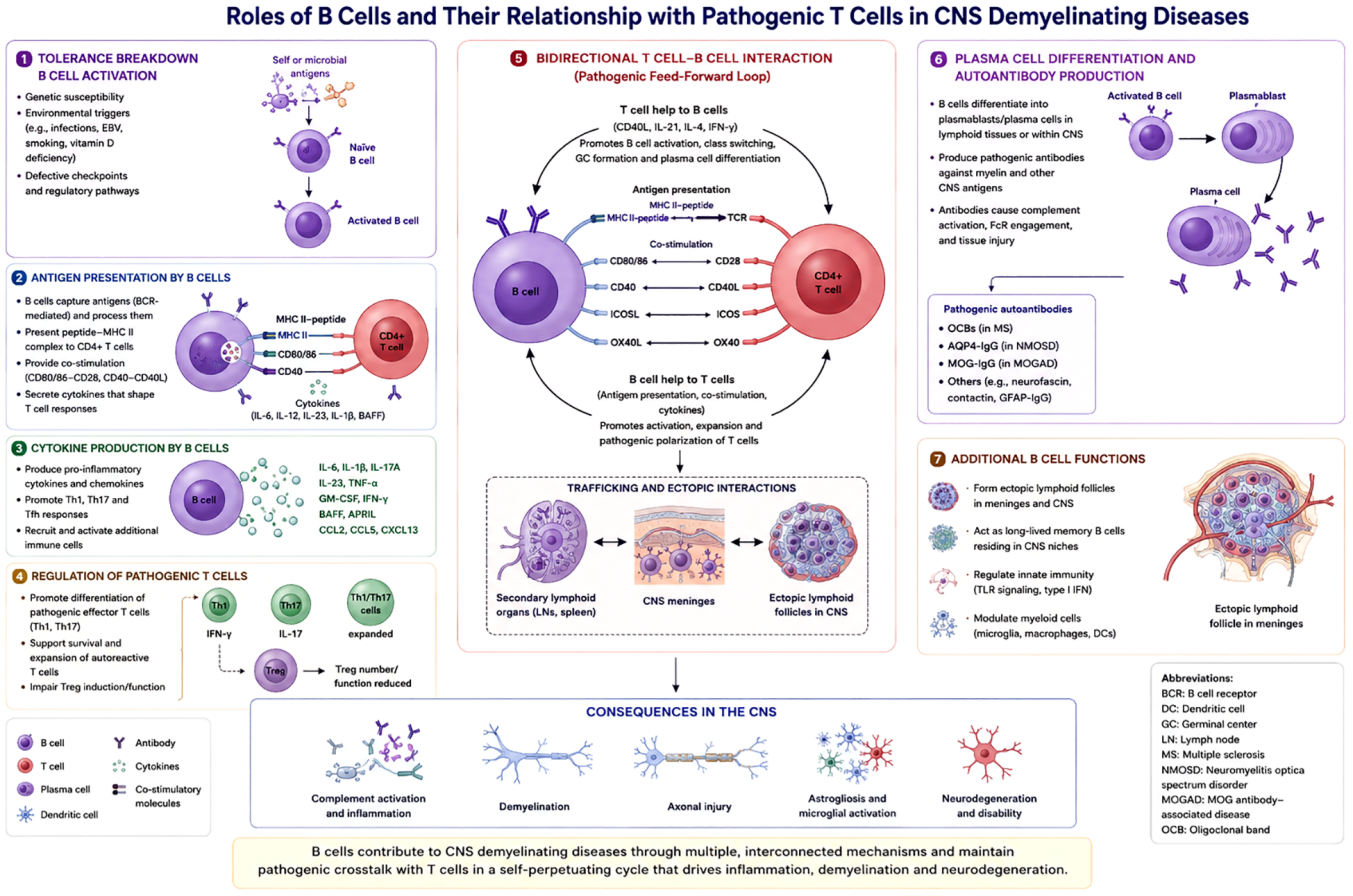
Mechanistic overview of B cells and their interactions with pathogenic T cells in CNS demyelinating diseases. B cells contribute to CNS demyelinating diseases through mechanisms extending beyond antibody production. Breakdown of immune tolerance permits activation of autoreactive B cells, which present antigens to CD4^+^ T cells via MHC class II and co-stimulatory pathways, including CD40–CD40L, CD80/86–CD28, ICOS–ICOSL, and OX40–OX40L. B cells also secrete pro-inflammatory cytokines that promote Th1, Th17, and Tfh responses while impairing regulatory T-cell function. Reciprocal T-cell help further drives B-cell activation, germinal center formation, class switching, and plasma-cell differentiation, establishing a pathogenic feed-forward loop. These interactions contribute to CNS-compartmentalized inflammation, ectopic lymphoid follicle formation, and production of pathogenic autoantibodies, including oligoclonal bands in MS, AQP4-IgG in NMOSD, and MOG-IgG in MOGAD. Collectively, these processes promote complement activation, demyelination, axonal injury, astrocytopathy, microglial activation, and neurodegeneration.

In this chapter, we review emerging concepts in B-cell biology relevant to immune-mediated demyelinating diseases, with a focus on mechanisms governing pathogenic and regulatory activities (see **Figure 1**). We examine how defects in B-cell tolerance, antigen presentation, cytokine signaling, and the contribution of B cells residing in the skull bone marrow shape CNS inflammation. We further discuss the implications of these findings for current and future therapeutic strategies. By integrating mechanistic and clinical perspectives, this chapter provides a framework for understanding B–cell–driven neuroinflammation and its relevance for precision immunomodulation.

## B-cell tolerance and CNS inflammation

B-cell tolerance comprises a multilayered set of immunological safeguards that prevent the survival and activation of autoreactive B cells, thereby limiting the production of pathogenic autoantibodies. Because B cells generate a highly diverse antigen receptor repertoire through stochastic recombination of variable (V), diversity (D), and joining (J) genes, the emergence of self-reactive clones is considered an inevitable consequence of normal B-cell development (23). However, B-cell development and activation are tightly regulated by central and peripheral tolerance checkpoints, which ensure immune competence while preventing autoimmunity (24).

Central tolerance occurs in the bone marrow, where immature B cells expressing high-affinity autoreactive B-cell receptors are eliminated or silenced through receptor editing (25, 26), clonal deletion (27) (28), or functional anergy (29). Peripheral tolerance mechanisms in secondary lymphoid organs regulate autoreactive B cells that escape central selection. These mechanisms control B-cell survival and activation through tightly regulated signals, including B-cell activating factor (BAFF), Fas/FasL interactions, and crosstalk with regulatory T cells (Tregs) (30). Within germinal centers, somatic hypermutation can generate *de novo* autoreactivity, requiring additional control by follicular helper T cells (Tfh) and follicular regulatory T cells (Tfr) to limit the selection and expansion of potentially pathogenic clones (31). Failure at any central or peripheral tolerance checkpoint allows the accumulation and activation of autoreactive B cells (32, 33).

Once tolerance is breached, B cells contribute to CNS pathology through multiple mechanisms (10, 34). These include the production of autoantibodies targeting CNS antigens, such as AQP-4 in NMOSD and MOG in MOGAD, as well as antibody-independent functions, including antigen presentation, costimulatory signaling, and cytokine production. Subsequently, these immunne activities of B cells can trigger CNS inflammation, demyelination, and neuronal injury (15, 35–37). Studies in human MS patients provide important insights into the nature of tolerance failure. Contrary to earlier expectations, central B-cell tolerance appears largely intact in most MS patients (32, 38). Instead, autoreactivity arises predominantly from defects in peripheral tolerance, resulting in the accumulation of autoreactive and polyreactive mature naïve B cells (38). These findings identify peripheral checkpoints as critical sites of pathogenic dysregulation in MS. Consistent with this model, genome-wide association studies implicate immune regulatory pathways across multiple lineages, including B cells, antigen-presenting cells (APCs), and Tregs (39). Notably, Tregs in MS exhibit impaired suppressive capacity and aberrant interferon-γ production, reflecting defective immune cross-talk that may further exacerbate pathogenic B-cell responses (40).

B-cell tolerance breakdown is not uniform across autoimmune diseases, even within CNS demyelinating diseases. Distinct patterns of tolerance disruption have been described in NMOSD and MOGAD, potentially reflecting differences in antigen specificity, disease chronicity, and immune architecture (41). In both conditions, central tolerance may be inherently limited, as developing B cells in the bone marrow are unlikely to encounter sufficient levels of CNS-restricted antigens to consistently induce deletion or receptor editing. Peripheral tolerance mechanisms also appear to be altered, although with potentially divergent outcomes. In MOGAD, tolerance disruption has been suggested to be episodic and context-dependent, based on observations such as fluctuating MOG-IgG titers and evidence of T cell–dependent, germinal center–associated B-cell activation. In contrast, NMOSD is more consistently associated with persistent B-cell autoreactivity and the presence of stable, high-affinity AQP4-IgG antibodies, which may reflect a more sustained loss of tolerance (42, 43). These observations suggest that MOGAD may involve a more dynamic and potentially reversible pattern of B-cell tolerance disruption, whereas NMOSD is more commonly associated with sustained, antibody-mediated autoreactive responses, although the extent to which these patterns differ mechanistically remains an area of ongoing investigation (44).

## Antibody-producing plasma cells

Plasma cell differentiation is initiated by antigen recognition through the B-cell receptor (BCR) (45). Upon encountering cognate antigen, B cells internalize and process antigenic peptides for presentation on major complex class II molecules (MHC II), enabling interaction with CD4^+^ T cells. Formation of a stable immunological synapse facilitates bidirectional signaling that promotes B-cell activation and T-cell help (46). Co-stimulatory signals, particularly CD40–CD40L interactions, drive B-cell proliferation, class-switch recombination, and differentiation into memory B cells or antibody-secreting plasma cells (47). In CNS demyelinating diseases, B-cell differentiation into plasma cells constitutes a critical pathogenic axis, although the contribution of antibodies to tissue injury varies substantially across diseases (15, 48).

### Plasma cells and compartmentalized immunoglobulin production in MS

A defining feature of humoral immune activation in MS is the presence of oligoclonal bands (OCBs) in the cerebrospinal fluid (CSF) (49). These clonally restricted immunoglobulins are typically absent from peripheral blood, reflecting compartmentalized intrathecal antibody production within the CNS (50). The persistence of OCBs indicates long-lived plasma cell survival and ongoing B-cell activation behind the blood–brain barrier (BBB), largely independent of systemic immune activity. Although the precise pathogenic role of intrathecal antibodies remains incompletely defined, immunoglobulin and complement deposition frequently co-localize within active MS lesions, suggesting that antibody-mediated mechanisms may contribute to demyelination and tissue injury (51).

OCBs in MS exhibit marked antigenic heterogeneity (52). Some antibodies recognize non-CNS antigens, including viral epitopes such as rubella, measles, and varicella-zoster virus, consistent with bystander activation or reactivation of memory B cells within the CNS (53). Others target CNS-associated antigens, including myelin components, astrocytes, and neuroglial structures, supporting a potential direct role in CNS autoimmunity (54). This diversity highlights the heterogeneity of plasma cell responses in MS and supports the concept that disease pathogenesis involves multiple overlapping immunopathogenic pathways, rather than being solely antibody-driven.

### Autoantibody roles in NMOSD and MOGAD

NMOSD represents a prototypical antibody-mediated CNS autoimmune disorder, in which a primary astrocytopathy is driven by AQP4-IgG autoantibodies (55). Because its clinical features overlap with MS, NMOSD was historically considered a subtype of MS. However, the identification of NMOSD-IgG by Lennon and colleagues in the early 2000s established a biomarker that reliably distinguishes NMOSD as a distinct disease entity (15, 56, 57). AQP4-IgG binds selectively to the astrocytic water channel AQP4, which is highly expressed on astrocytic endfeet but absent from oligodendrocytes, defining astrocytes as the principal cellular target (58–60). In active lesions, AQP4-IgG deposits alongside immunoglobulin and complement, leading to loss of AQP4 and glial fibrillary acidic protein (GFAP), reflecting antibody- and complement-mediated astrocyte destruction.

Although highly sensitive assays detect AQP4-IgG in the majority of patients, 10–40% of individuals with NMOSD lack these antibodies (61). In a subset of seronegative cases, pathogenic autoantibodies targeting MOG, a surface protein on the outer myelin sheath, are present (62). MOGAD is characterized by primary myelin injury without astrocytopathy, clinically presenting with optic neuritis, transverse myelitis, acute disseminated encephalomyelitis (ADEM), and, less commonly, cortical encephalitis (44). Unlike NMOSD, MOGAD often follows a monophasic course in 40–50% of patients, while relapsing cases are associated with persistent high MOG-IgG titers (63, 64). Pediatric and adult-onset MOGAD patients exhibit similar annualized relapse rates, in contrast to MS, in which pediatric-onset disease is generally more inflammatory (65). Infectious prodromes occur more frequently in MOGAD than in NMOSD, suggesting environmental triggers in disease onset or relapse (66).

B-cell biology differs markedly between these disorders. In NMOSD, AQP4-specific B cells are activated in the periphery, differentiate into plasma cells, and traffic across the BBB to mediate astrocyte injury (19). Although serum AQP4-IgG titers do not always correlate with disease activity, the presence of CNS-infiltrating B cells and plasma cells underscores the pathogenic significance of antibody deposition and B-cell migration (15, 16). In MOGAD, peripheral MOG-specific B cells similarly differentiate into plasma cells capable of producing pathogenic antibodies; however, the relative ineffectiveness of anti-CD20 therapies suggests a prominent role for long-lived plasma cells or antibody-independent mechanisms (67). Coexisting autoantibodies are uncommon, and epitope spreading is not well established; however, antibodies such as anti-GRP78 (78-kDa glucose-regulated protein) may contribute to BBB disruption during acute attacks (68). While AQP4-IgG–positive NMOSD is characterized by a high-affinity, antibody-driven astrocytopathy, MOGAD exhibits greater heterogeneity, involving diverse B-cell and antibody-mediated mechanisms (69, 70). Pathogenic mechanisms of MOG antibodies are thought to include opsonization of myelin, complement activation, antibody-dependent cellular cytotoxicity, and induction of intracellular signaling cascades (71, 72).

### B cells as professional APCs in CNS inflammation

B cells are professional APCs that play a central role in shaping T cell responses in both peripheral and CNS immune compartments (73–75). Unlike myeloid APCs, which ingest antigens indiscriminately, B cells can recognize even low concentrations of antigens specifically through their BCRs and constitutively express MHC II, along with co-stimulatory proteins such as CD40, CD80, and CD86 (76, 77). This specialized machinery enables B cells to internalize natively folded “conformational” protein antigens, process them into linear peptides, and efficiently present them to antigen-specific CD4^+^ T cells. Consequently, B cells are particularly effective at activating T cells with shared antigen specificity, which in turn promotes their differentiation into memory B cells and antibody-producing plasma cells (78, 79).

### MS and EAE

The critical role of B cells as antigen-presenting cells in CNS autoimmunity is well demonstrated in murine models (80, 81). In experimental autoimmune encephalomyelitis (EAE) (82), a model of human inflammatory CNS demyelinating disorders, selective ablation of MHC class II on B cells renders mice resistant to disease induction, highlighting the substantial role of B cells as antigen-presenting cells. Efficient priming of naïve T cells requires not only peptide presentation via MHC class II but also the provision of appropriate co-stimulatory signals. For example, strong engagement of CD40 on B cells with CD40 ligand (CD40L) on T cells is required for generating pro-inflammatory effector T cells in both human and mouse cells (77, 83), In contrast, weaker CD40–CD40L interactions favor the induction of regulatory T cells in mouse models (84). Complete blockade of murine CD40–CD40L signaling can even prevent EAE, highlighting the critical role of co-stimulation in shaping T-cell outcomes (77, 85, 86).

Clinical evidence from MS patients supports these mechanistic insights. B cells from individuals with active MS display elevated expression of CD40, MHC class II, and CD80 compared with healthy controls, indicating heightened antigen-presenting potential (87). Evidence from both peripheral blood and CNS compartments indicates that B cells in MS display chronic activation and a shift toward antigen-experienced memory subsets, reflecting their active role in disease processes. Functional assays show that B cells from relapsing–remitting MS patients can induce pathogenic CD4^+^ T helper 1 (Th1) proliferation and IFN-γ production in ex vivo settings (88). Collectively, these findings indicate that B-cell antigen-presenting activity promotes the activation of CNS-infiltrating T cells and sustains disease inflammation, highlighting a prominent cell-mediated role for B cells in MS pathogenesis that is independent of their humoral functions.

### NMOSD

In NMOSD, pathogenic interactions between B cells and T cells are increasingly recognized as pivotal drivers of disease pathogenesis (89). Circulating B cells from NMOSD patients exhibit altered activation states, skewed memory phenotypes, and dysregulated cytokine production, which collectively enhance their antigen-presenting capacity and promote pathogenic T cell responses (90, 91). CD40–CD40L interactions, along with cytokines such as IL-21, not only promote B cell activation, differentiation, and AQP4 antibody production (90), but also enhance B cell–mediated antigen presentation and cognate T cell activation. Indeed, B cells are thought to act as antigen-presenting cells for AQP4, priming autoreactive CD4^+^ T cells and driving their differentiation into T helper 17 cells (92). Th17 cells, in turn, provide reciprocal signals that reinforce B cell maturation and differentiation into AQP4 antibody–producing plasma cells, creating a pathogenic feed-forward loop. Consistent with their enhanced antigen-presenting capacity, highly self-reactive, antibody-producing double-negative CD11c^hi^ T-bet^+^ B cells, characterized by elevated APC potential, have been identified in human studies across multiple autoimmune diseases (93, 94). In NMOSD, the frequency of CD11c^hi^IgD^-^CD27^-^ B cells is elevated compared with healthy controls and correlates with increased brain atrophy and greater disease severity (95). Immunophenotypic analyses in NMOSD patients reveal increased frequencies of switched memory B cells and plasmablasts, accompanied by a reduction in naïve B cells, reflecting an enrichment of antigen-experienced B cells with heightened antigen-presenting capacity (96).

### MOGAD

Although MOGAD is defined by the presence of pathogenic antibodies, accumulating evidence indicates a prominent role for cellular immune mechanisms, particularly antigen-specific T cell responses, in disease initiation and relapse phases. B cells that recognize native, conformational MOG via the BCR are uniquely equipped to internalize and process MOG and present MOG-derived peptides to CD4^+^ T cells, thereby selectively activating cognate T cell populations (97). Such B cell–T cell interactions are likely critical in MOGAD (89), where relapse patterns and therapeutic responsiveness differ from NMOSD, suggesting distinct immunoregulatory mechanisms (44, 98–100). Consistent with this concept, the variable efficacy of B–cell–depleting therapies in MOGAD implies that qualitative differences in B-cell APC function, rather than antibody production alone, may influence disease outcomes (101).

Beyond direct antibody-mediated effects, cellular immune responses play a key role in shaping MOGAD pathogenesis. The activation and differentiation of MOG-specific B and T cells are essential for MOG-IgG production, and preceding infections, reported in 20–57% of cases, are thought to trigger disease by disrupting immune tolerance through mechanisms such as molecular mimicry, bystander activation, or exposure of normally sequestered MOG antigens within the CNS (97). Supporting a role for pathogenic T cell responses, stimulation of peripheral blood mononuclear cells (PBMCs) from MOGAD patients with recombinant human MOG protein induces increased Th2 and Th17 responses. In addition, dysregulation of follicular T cell subsets, marked by increased Tfh cells and reduced Tfr cells during clinical attacks, may further promote aberrant B cell activation, antigen presentation, and sustained autoantibody production (102, 103).

## B cells in CNS inflammation: cytokine secretion and immunoregulation

### MS and EAE

B cells contribute to CNS inflammation not only through antigen presentation but also by secreting cytokines that modulate immune responses. Effector and memory B cells release pro-inflammatory mediators, including IL-6, TNF-α, and GM-CSF, which activate microglia, astrocytes, and myeloid cells, thereby amplifying demyelination and neuroinflammation in both human studies and mouse models (11, 15, 104, 105). In contrast, Bregs produce anti-inflammatory cytokines such as IL-10, IL-35, and TGF-β, limiting autoreactive T cell activation and restraining excessive inflammation (8, 9). In the animal model of EAE, IL-10–producing Bregs are essential for controlling CNS inflammation, as their absence exacerbates disease. Consistently, adoptive transfer of wild-type, but not IL–10–deficient-B cells, ameliorates pathology (106). Similar regulatory functions have been observed in humans, and evidence suggests they may be impaired in MS and NMOSD, underscoring their therapeutic relevance (107, 108).

Cytokine secretion complements B cell co-stimulatory signaling, as T cell activation is shaped not only by direct cell–cell interactions but also by the surrounding cytokine milieu. For example, B–cell–derived IL-6 promotes Th17 differentiation while inhibiting regulatory T cell development in both human and murine systems (109, 110). In B cell–dependent EAE models, B cell-specific IL-6 deficiency reduces the Th17 response and ameliorates disease severity (105). In MS, peripheral B cells display an abnormal pro-inflammatory profile, with elevated secretion of IL-6, lymphotoxin-α, and TNF-α alongside reduced IL-10 (111). Memory B cells from MS patients often co-express GM-CSF, IL-6, and TNF-α, reflecting enhanced pathogenic potential. Therapeutic B-cell depletion not only reduces B-cell numbers but also decreases pro-inflammatory IL-6 production by macrophages, underscoring the role of B cells in shaping innate immune responses (111). B cells, therefore, act as both drivers and modulators of CNS autoimmunity (112).

### NMOSD

In NMOSD patients, Breg function appears markedly impaired, with reduced frequencies of IL-10–producing Bregs and diminished IL-10 secretion capacity. This deficit likely contributes to unchecked Th1 and Th17 responses and exacerbated CNS inflammation (113). Therapeutic blockade of IL-6 signaling significantly reduces relapse rates in NMOSD, supporting a central role for B–cell–driven cytokine pathways in disease pathogenesis (114). Accordingly, NMOSD is characterized by a skewing toward pro-inflammatory, plasmablast-rich B cell responses, reflecting both dysregulated cytokine secretion and enhanced antigen-presenting activity.

### MOGAD

In MOGAD, emerging evidence suggests that dysregulation of cytokine-producing B cell subsets contributes to disease pathogenesis beyond MOG-IgG production alone. Although the immunopathological role of B-cell–derived cytokines in MOGAD remains incompletely defined, current evidence suggests a distinct balance between pathogenic and regulatory B-cell functions (115). Elevated levels of Th17-related cytokines, including IL-6, have been detected in the CSF of MOGAD patients during acute attacks (116). B cells from MOGAD patients exhibit activation profiles indicative of prior antigen experience and elevated production of IL-6 and TNF-α, which may drive encephalitogenic T cell responses and activate myeloid cells within the CNS (117). Breg populations appear relatively preserved in some MOGAD cohorts compared with MS and NMOSD, potentially contributing to the typically favorable clinical recovery and responsiveness to steroid therapy in these patients (118). Peripheral B cell expansion has been observed in MOGAD patients without correlation to MOG-IgG titers, while detailed immunophenotyping reveals an expansion of memory B cells and Tfh cells, accompanied by a reduction in regulatory B cells (115).

Importantly, heterogeneity in B cell cytokine profiles likely underlies clinical variability in both NMOSD and MOGAD. NMOSD is characterized by a skewing toward pro-inflammatory, plasmablast-rich B cell responses, whereas MOGAD may display a more dynamic balance between effector and regulatory B cell subsets. These differences may help explain variability in treatment responses, including the inconsistent efficacy of B–cell–depleting therapies across antibody-mediated demyelinating disorders.

## B cells in CNS niches and their roles in demyelinating diseases

Under physiological conditions, lymphocyte entry into the CNS is tightly restricted by specialized barriers, including the BBB, the blood–CSF barrier, and the meningeal vasculature. We define ‘CNS immune niches’ as anatomically and functionally specialized microenvironments at CNS borders, encompassing the meninges, perivascular spaces, choroid plexus, CSF, and adjacent skull and vertebral bone marrow (119). CNS immune niches can support B cell persistence, activation, and differentiation independently of classical peripheral lymphoid organs. During neuroinflammation, these niches become permissive, allowing B cells to traffic into CNS compartments via multiple entry routes, where local cues influence their survival, activation, and the balance between pathogenic and regulatory functions (104, 120, 121).

In demyelinating diseases such as MS, NMOSD, and MOGAD, CNS niche B cells undergo pronounced phenotypic and functional reprogramming. Inflammatory cytokines, pattern-recognition receptor ligands, and chemokine gradients promote class switching and enhanced effector functions in B cells (122, 123). Upregulation of CXCR5 and CCR7 enhances B cell responsiveness to CXCL13 and CCL19/CCL21, promoting migration toward CNS border regions and supporting the formation of meningeal immune infiltrates and tertiary lymphoid structures (TLS), particularly in progressive MS (124). Within these niches, B cells secrete pro-inflammatory cytokines, including TNF-α, GM-CSF, and IL-6 that activate microglia and astrocytes, thereby amplifying neuroinflammation and demyelination (104). The transition of CNS niche B cells from homeostatic regulators to pathogenic effectors also depends on interactions with autoreactive CD4^+^ T cells, for example, these T cells provide CD40-dependent costimulation that drives class switching and plasma cell differentiation (83). Disease-associated epigenetic remodeling enhances expression of transcriptional regulators such as Blimp-1 and IRF4, promoting both antibody production and pro-inflammatory cytokine secretion (125). Repeated antigen encounters within CNS niches further drive clonal B cell expansion and tissue injury.

### Skull bone marrow: CNS-proximal B-cell reservoir

The skull bone marrow (SBM) is a CNS-adjacent immune reservoir with unique anatomical and functional features (126). Unlike long bones, the skull develops via intramembranous ossification, supporting specialized hematopoiesis and lifelong vascular remodeling (127). SBM hosts hematopoietic stem cells (HSCs) that give rise to myeloid and lymphoid lineages, including B cells, which traffic to the meninges via skull-meningeal connections (SMCs), vascular channels linking the inner skull cortex to dural vasculature (128). Mouse studies demonstrate 20–25 μm-wide channels, while human imaging suggests even larger conduits capable of substantial immune cell trafficking (129, 130).

SBM-derived B-lineage cells have been shown in experimental models to populate meningeal niches and may contribute to CNS immune homeostasis (131). These cells preferentially produce regulatory cytokines such as IL-10, suppress myeloid and T-cell activation, and generate minimal antibody output, largely restricted to natural antibodies involved in debris clearance conditions (132). In MS models, autoreactive T cells infiltrate SBM, stimulating myelopoiesis and exacerbating CNS inflammation (133). Human TSPO-PET imaging confirms skull marrow activation in both relapsing and progressive MS, highlighting its role in shaping the CNS immune landscape (134).

Single-cell RNA sequencing and parabiosis studies indicate that meningeal B cells largely resemble SBM B cells across developmental stages, whereas circulating blood and splenic B cells predominantly display a mature phenotype (135, 136). These observations raise the possibility that a proportion of meningeal B cells may arise from CNS-adjacent bone marrow niches, although this remains to be fully established, particularly in humans. Beyond myeloid support, SBM contributes to CNS tolerance by supplying immature and mature B cells to dural tissue, where interaction with CNS-derived antigens such as MOG facilitates negative selection of autoreactive clones. Disruption of this process may allow autoreactive lymphocytes to persist, exacerbating autoimmune diseases such as MS (137–140).

### Meninges: immune hub and site of TLS formation

The meninges comprise three protective layers including dura mater, arachnoid mater, and pia mater, that are highly vascularized and functionally integrated with lymphatic drainage, paravascular glymphatic flow, and immune surveillance (141). The dura mater is fibrous and robust, containing large arteries, veins, and a lymphatic network that facilitates CSF drainage into the lymphatic system (142, 143). Both paravascular glymphatic flow and meningeal lymphatic CSF drainage undergo dynamic changes across the lifespan, playing essential roles in fluid clearance, immune surveillance, and CNS homeostasis (144, 145). Meningeal immunity reflects the coordinated integration of these systems, encompassing glymphatic transport, lymphatic vessels, resident immune populations, and cytokine signaling networks (146, 147). Through continuous sampling of CSF, meningeal immune cells monitor brain homeostasis, detect pathological cues, and initiate appropriate immune responses, thereby serving as a critical interface that limits pathogen entry into the CNS (143). The meninges harbor a diverse repertoire of immune cells, including T cells, B cells, neutrophils, dendritic cells (DCs), macrophages, and mast cells (148, 149). CSF communicates with skull bone marrow niches through dura-skull channels, creating a feedback loop that recruits immune cells to the meninges under physiological and pathological conditions (135).

During neuroinflammation, meninges serve as a critical site for the initiation and organization of tertiary lymphoid structures (TLSs) (150). TLSs are ectopic lymphoid aggregates containing organized B and T cell zones, follicular dendritic cells, and stromal support networks (151). In EAE models, TLSs form preferentially in the leptomeninges and dura near subpial cortical lesions (150), while postmortem MS studies demonstrate TLSs containing diverse immune populations, including B cells, T cells, dendritic cells, macrophages, plasma cells, and stromal cells (152, 153). These structures provide local niches for antigen presentation, T and B cell activation, and germinal center-like responses, sustaining chronic inflammation and driving cortical pathology (154). B cells within TLSs are largely naïve or early-stage, but local maturation and class switching can occur. TLS density correlates with lesion severity, white matter damage, and clinical progression (155).

Of note, CXCL13 expression within TLSs recruits and retains CXCR5^+^ lymphocytes, particularly B cells and Tfh cells, sustaining organized antigen-driven immune responses (152, 156–158). BAFF expression within TLSs supports B cell survival and activation, while RORγt^+^ Th17 cells contribute to TLS formation and IL-17–driven local inflammation (159–161). Together, the meninges function as active immune hubs, integrating surveillance, hematopoietic crosstalk, and local adaptive immunity (162).

### CSF: intrathecal niche and T-B crosstalk

The CSF is a plasma-like CNS compartment essential for brain homeostasis, providing nutrient delivery, waste clearance, and mechanical protection. It is produced primarily by the choroid plexus within the lateral, third, and fourth ventricles, with a smaller contribution from ependymal cells lining the ventricles (163). CSF circulates through the ventricular system into the subarachnoid space and is eventually reabsorbed into venous circulation via arachnoid villi. Importantly, CSF functions as a central component of the glymphatic system, facilitating clearance of brain-derived molecules via efflux to the parasagittal dura and drainage through meningeal lymphatic vessels, thereby connecting CNS compartments with peripheral immune surveillance (137, 138, 164). Recent studies also demonstrate that CSF can access skull bone marrow niches, modulating myelopoiesis and the subsequent egress of immune cells to the meninges under both physiological and neuroinflammatory conditions (139, 165).

CSF harbors a tightly regulated immune environment that supports CNS homeostasis. In healthy individuals, the majority of CSF immune cells are CD4^+^ T cells (~50%), followed by CD8^+^ T cells (~18%), with smaller populations of B cells and monocytes (166). During neuroinflammation, as observed in MS, the CSF immune landscape undergoes profound changes. There is an enrichment of clonally expanded B cells, cytotoxic CD4^+^ T cells, Tregs, and myeloid dendritic cells (mDCs), reflecting enhanced local antigen presentation and intrathecal inflammation. Monocytes in the CSF exhibit transcriptional profiles resembling CNS border-associated macrophages, suggesting functional specialization for CNS immune responses (156, 167).

Beyond serving as a conduit for immune trafficking, the CSF functions as an active niche for pathogenic T–B cell interactions. In MS, aberrant engagement between Tfh cells and B cells promotes sustained intrathecal immune responses (168). Expanded B cell clones within the CSF arise through both peripheral recruitment and local maturation, consistent with ongoing antigen-driven inflammation. Within this environment, B cells can differentiate into plasmablasts and plasma cells that produce antibodies targeting CNS antigens such as myelin basic protein (MBP), MOG, or AQP4, driving complement activation, antibody-dependent cytotoxicity, and lesion formation. Both human neuropathological studies and animal models support the existence of long-lived, compartmentalized plasma cell populations within the CSF that are relatively resistant to systemic immunosuppression and contribute to chronic disease activity and relapses (134, 169).

## Insights from CNS immune niches

- Overlap between CSF and meningeal BCR repertoires may correlate with ongoing disease activity despite peripheral B-cell depletion (170).
- CSF CXCL13 levels reflect intrathecal B-cell recruitment and TLS activity, serving as a biomarker for disease progression and compartmentalized immune activity (171), as CXCL13 seems to be the major determinant for B cell recruitment to the CNS compartment in different neuroinflammatory diseases in both human studies and mouse models (157, 172–174).
- Advanced imaging detecting meningeal inflammation or TLS-like structures predicts progression and treatment resistance (150, 155). Integrating CNS-focused biomarkers with targeted interventions offers a path toward precise and durable control of demyelinating disease (175).

## B-cell–targeted therapy in MS, NMOSD, and MOGAD

### MS

The therapeutic success of CD20-depleting monoclonal antibodies has transformed MS treatment (176). These agents selectively eliminate circulating and secondary lymphoid tissue B cells while sparing plasma cells and early B-cell progenitors, thereby preserving baseline humoral immunity (4). Clinical trials have supported that B-cell depletion leads to rapid and profound reductions in relapse rates (13, 21, 177), new MRI lesion formation, and disability progression in both relapsing and progressive forms of MS (178). Notably, the speed of clinical efficacy suggests that suppression of B-cell antigen presentation and cytokine signaling, rather than reduction of autoantibodies, might be the dominant mechanism of benefit (51, 179).

B-cell therapies have also reshaped our understanding of progressive MS. In primary and secondary progressive disease, B-cell–rich meningeal inflammation is associated with cortical demyelination and neurodegeneration (153). The ability of B-cell depletion to slow disability accumulation in these populations highlights the contribution of compartmentalized, chronic immune activation to progressive pathology. However, incomplete efficacy in advanced disease stages suggests that CNS-resident immune cells and neurodegenerative mechanisms may become partially independent of peripheral immune input over time (126, 180).

Despite their effectiveness, B-cell–targeted therapies raise important questions regarding long-term immune modulation. Sustained therapy can impair vaccine responses and increase susceptibility to infections, underscoring the need to balance durable disease control with preservation of immune competence (181).

### NMOSD and MOGAD

The differentiation state of B cells has important therapeutic implications for NMOSD and MOGAD, Anti-CD20 therapies deplete pre-B cells through memory B cells but largely spare plasmablasts and long-lived plasma cells, whereas anti-CD19 therapies target a broader range of B-cell developmental stages, including antibody-secreting cells. Because plasmablasts and plasma cells are the primary source of AQP4 antibodies, the efficacy of B-cell–directed therapies in NMOSD may depend critically on the depth and specificity of depletion (90). Clinically, anti-CD20 therapies are highly effective in reducing relapse rates in NMOSD, while anti-CD19 therapies achieve deeper B-cell depletion and can reduce relapses even in rituximab-refractory patients.

In contrast, in MOGAD, anti-CD20 therapy reduces circulating B cells but often fails to prevent relapses, which may persist due to long-lived plasma cells or antibody-independent mechanisms. Persistent MOG-IgG strongly correlates with disease activity, and adjunctive therapies such as corticosteroids or intravenous immunoglobulin are frequently required. These observations highlight that B-cell depletion alone is often insufficient for durable disease control in MOGAD, emphasizing the need for alternative or combination strategies to target plasma cells or downstream effector pathways.

## Limitations and resistance to anti-CD20 therapies

Anti-CD20 therapies used in MS and other demyelinating diseases effectively target B cells; however, long-term B-cell depletion is associated with several important limitations. Anti-CD20-depleting therapies are associated with reductions in serum immunoglobulin (Ig) levels, an increased risk of infections, and diminished responses to vaccination (182).

Notably, hypogammaglobulinemia may develop months to years after initiation of anti-CD20 therapy (183). Although plasmablasts and plasma cells that produce IgG and IgM do not express CD20, one proposed mechanism is that anti-CD20 agents impair the regenerative capacity of B-cell populations (184). In addition, long-lived memory plasma cells that persist after B-cell depletion may not be entirely self-sustaining and may require replenishment from CD20-expressing B-cell progenitors, thereby contributing to progressive declines in immunoglobulin levels over time (185). Reduced serum immunoglobulin levels during anti-CD20 treatment can compromise host immune defense, placing patients at increased risk for infections, including severe respiratory, cutaneous, and opportunistic infections that may require hospitalization (183) and, in some cases, may be fatal (186). Among immunoglobulin subclasses, IgG has the longest serum half-life and the highest circulating concentration and plays a critical role in pathogen clearance, complement activation, and immunological memory. This may explain why IgG deficiency is generally more detrimental to immune defense than IgM deficiency. Individuals receiving B-cell-depleting therapies may also exhibit impaired humoral responses to vaccination, including reduced responsiveness to SARS-CoV-2 vaccines (187).

Resistance to anti-CD20 therapies and subsequent disease relapse remain important clinical challenges, partly because anti-CD20 agents do not target plasma cells, which can continue producing autoantibodies and thereby contribute to persistent disease activity despite treatment. In addition, several mechanisms may underlie resistance or suboptimal therapeutic response:

- Incomplete tissue depletion: B cells may persist within lymphoid organs or the CNS, serving as reservoirs of ongoing immune activation (188).
- Rapid B-cell repopulation: Early B-cell reconstitution may result in renewed clinical and radiological disease activity (189).
- Antigenic modulation: CD20 downregulation or internalization, particularly following treatment with type I antibodies such as rituximab, may reduce therapeutic targeting (190).
- CD20-expressing T cells: A subset of CD20^+^ T cells may persist within the cerebrospinal fluid and contribute to ongoing inflammation.
- Fcγ receptor polymorphisms: Genetic variation in FcγRIIIA may impair antibody-dependent cellular cytotoxicity (ADCC), thereby reducing the efficiency of B-cell depletion (191, 192).

As limitations and resistance to B-cell-depleting therapies become increasingly recognized, emerging therapeutic strategies aim to selectively modulate pathogenic B-cell functions without inducing broad immune depletion. Significant progress has been made in the development of target-specific therapies for autoimmune diseases, particularly those directed against distinct B-cell populations and signaling pathways. These approaches include next-generation anti-CD20 therapies (e.g., type II anti-CD20 antibodies), targeting of specific B-cell subsets such as CD38 and CD22, modulation of intracellular signaling through Bruton’s tyrosine kinase (BTK) inhibitors (177, 193), co-stimulation blockade via anti-CD40 therapies, inhibition of B-cell survival factors such as BAFF (194), and the use of bispecific antibodies, including obexelimab. More recently, cellular immunotherapies such as anti-CD19 chimeric antigen receptor (CAR) T-cell therapies have emerged as promising approaches for refractory autoimmune diseases (195).

## Conclusions and future perspectives

In this conclusion, we have summarized the multifaceted roles of B cells in CNS demyelinating diseases and highlighted several key unresolved questions.

### Antibody-dependent and antibody-independent roles of B cells in CNS pathology

Evidence for antibody-dependent mechanisms includes the presence of oligoclonal IgG bands in cerebrospinal fluid, reflecting sustained intrathecal B-cell activation (196, 197). Meningeal B-cell aggregates and follicle-like structures, particularly in progressive disease, are associated with cortical demyelination and neurodegeneration (141, 150, 153). In addition, complement and immunoglobulin deposition in a subset of active lesions supports antibody-mediated injury, consistent with “pattern II” pathology (72, 198). Therapeutically, the efficacy of anti-CD20 B-cell–depleting therapies supports a central role for B cells despite persistence of long-lived plasma cells, suggesting mechanisms beyond antibody production. The absence of consistent target antigens further raises the possibility that oligoclonal responses may reflect bystander or antiviral immune activity rather than myelin-specific autoimmunity.

Substantial evidence also supports antibody-independent mechanisms. Active lesions are enriched in CD8^+^ T cells, which correlate with axonal damage and may directly injure oligodendrocytes via MHC class I–restricted cytotoxicity (2). Microglia and macrophages further contribute through phagocytosis, cytokine release, and oxidative stress (44). Notably, in pattern I lesions and progressive MS, pathology often occurs with minimal humoral immune deposition, supporting predominantly antibody-independent inflammation (198, 199).

Rather than representing opposing mechanisms, antibody-dependent and antibody-independent B-cell functions can be viewed as complementary components of a broader and dynamic immune network. These processes may coexist, cooperate, or dominate at different stages of disease. Accordingly, a comprehensive understanding of CNS demyelinating disease requires integrating all functional aspects of B cells to capture the full complexity and temporal evolution of pathology.

### CNS-restricted versus peripheral immune contributions in disease progression

In progressive stages of CNS demyelinating diseases, CNS-resident immune cells become increasingly dominant. In this context, inflammatory activity is largely confined to the CNS, with chronic microglial activation and ongoing neurodegeneration persisting even in the relative absence of overt adaptive immune infiltration (104, 155). This suggests that neurodegenerative processes may become partially self-sustaining and progressively less dependent on peripheral immune activity (200).

### T–B cell interactions as a central pathogenic axis in CNS autoimmunity

Although B cells are central to disease pathogenesis and therapeutic response, T cells remain equally important and cannot be considered in isolation. This supports the view that disease activity is not driven by a single dominant immune subset, but rather by coordinated and interdependent B–T cell interactions. Emerging evidence indicates that such interactions represent a critical pathogenic axis.

Beyond antibody production, B cells function as antigen-presenting cells that activate CD4^+^ T cells via MHC class II and co-stimulatory signaling (82). In turn, follicular helper T (Tfh) cells promote B-cell maturation, germinal center formation, and antibody production, establishing a self-amplifying feedback loop (201). Key pathways, including CD40–CD40L (85), ICOS–ICOSL (202), and OX40–OX40L (203), regulate this interaction and represent potential therapeutic targets.

Particularly, unconventional lymphocyte populations may further bridge these immune compartments. Dual-expresser lymphocytes (DEs), which co-express T-cell receptor αβ and immunoglobulin, have been identified in patients with MS (204). These cells are enriched in blood and CSF, respond to myelin antigens, express CD20, and are depleted by anti-CD20 therapy, implicating them in disease pathogenesis and highlighting them as potential therapeutic targets.

In summary (see **Figure 1**; **Table 1**), this review highlights the multifaceted roles of B cells in CNS demyelinating diseases, including multiple sclerosis (MS), neuromyelitis optica spectrum disorder (NMOSD), and MOG antibody-associated disease (MOGAD). Beyond antibody production, B cells contribute to disease pathogenesis through antigen presentation, co-stimulatory signaling, cytokine secretion, and dynamic interactions with pathogenic T cells (**Table 2**). The review discusses mechanisms of B-cell tolerance breakdown, CNS-compartmentalized immunity, and emerging concepts such as dual-expresser lymphocytes that may bridge humoral and cellular immunity. In addition, it examines the therapeutic implications of B-cell–targeted therapies, including anti-CD20 agents, treatment resistance, and emerging precision immunotherapies. Collectively, these findings support an integrated model of neuroinflammation in which B-cell functional heterogeneity and T–B cell interactions play central roles in disease progression and therapeutic response.

**Table 1:** Comparative overview of B-cell biology across CNS demyelinating diseases.

Disease	Dominant B-cell mechanisms	Key biomarkers	Primary lesion target	Therapeutic implications
MS	•Antigen presentation to CD4^+^ T cells •Pro-inflammatory cytokine secretion (IL-6, GM-CSF, TNF-α) •Meningeal TLS formation •Compartmentalized intrathecal plasma cells (OCBs) •Antibody-independent mechanisms dominant	•CSF oligoclonal bands (OCBs) • Elevated CXCL13 in CSF •Expanded memory B cells •CD40, MHC II upregulation •BCR clonality overlap (CSF– meninges)	•Myelin and axons •Secondary cortical neurodegeneration •Subpial demyelination (progressive MS)	•Strong response to anti-CD20 therapy •Efficacy driven largely by APC/cytokine suppression •BTK inhibition as compartmental modulation strategy •Plasma cells relatively resistant to CD20 depletion
NMOSD	•High-affinity AQP4-IgG production •Complement activation •Plasmablast expansion •B–T cell feed-forward loop (Th17 skewing)	•Serum AQP4-IgG •Elevated IL-6 •Increased CD11c^+^ double-negative B cells •Plasmablast expansion	•Astrocytes (AQP4^+^ endfeet) •Secondary demyelination •Necrotic lesions	•Highly responsive to anti-CD20 •Anti-CD19 (inebilizumab) deeper depletion •IL-6 receptor blockade effective •Complement inhibition (eculizumab) directly targets effector pathway
MOGAD	•MOG-IgG–mediated myelin injury •Antigen presentation to MOG-specific T cells •Th17/Th2 responses •Mixed antibody-dependent and cellular mechanisms	•Serum MOG-IgG (titer correlates with relapse risk) •CSF IL-6 during attacks •Memory B cell expansion •Tfh/Tfr imbalance	•Oligodendrocytes and outer myelin sheath •Optic nerve, spinal cord, •ADEM-like lesions	•Variable response to anti-CD20 •Plasma cell persistence likely relevant •Steroid responsiveness common •IVIG often effective •Potential need for plasma cell or combination therapy

**Table 2:** Antibody-independent roles of B cells in CNS demyelinating diseases.

Antibody-independent function	Key mechanisms	Evidence in MS / NMOSD / MOGAD	Pathogenic consequences	Therapeutic relevance
Antigen presentation (MHC class II)	B cells present autoantigens to CD4^+^ T cells and provide co-stimulation	Strong T-cell activation in CSF and CNS lesions; B-cell depletion reduces T-cell activation	Drives expansion and persistence of autoreactive T cells	Explains efficacy of anti-CD20 therapies beyond antibody removal
Co-stimulatory signaling	CD40–CD40L, ICOS–ICOSL, OX40–OX40L interactions with T cells	Upregulated co-stimulatory molecules in CNS immune niches	Sustains chronic T–B cell activation loop and T-cell effector function	Potential targets for next-generation immunotherapies
Cytokine production (pro-inflammatory)	Secretion of IL-6, TNF-α, GM-CSF, lymphotoxin	Pro-inflammatory B-cell subsets identified in blood, CSF, and lesions	Promotes Th1/Th17 polarization, microglial activation, and neuroinflammation	Supports selective targeting of pathogenic B-cell subsets
Cytokine production (regulatory)	IL-10–producing regulatory B cells (Bregs)	Reduced Breg function reported in active disease	Loss of immune regulation and increased inflammation	Suggests therapeutic potential of restoring regulatory B-cell balance
Interaction with T cells	Bidirectional B–T crosstalk via antigen presentation, cytokines, and co-stimulation	Tfh expansion; B-cell depletion reduces T-cell activation	Drives self-amplifying B–T feedback loop sustaining chronic inflammation	Targeting T–B interaction axis may reduce disease activity
Formation of ectopic lymphoid structures	Organization of meningeal follicle-like structures with T cells and myeloid cells	Observed in progressive MS meninges	Creates CNS “immune niches” sustaining chronic inflammation	Highlights CNS compartment as autonomous immune site
